# A Chaperone for the Stator Units of a Bacterial Flagellum

**DOI:** 10.1128/mBio.01732-19

**Published:** 2019-08-06

**Authors:** Deborah A. Ribardo, Brittni R. Kelley, Jeremiah G. Johnson, David R. Hendrixson

**Affiliations:** a Department of Microbiology, University of Texas Southwestern Medical Center, Dallas, Texas, USA; b Department of Microbiology, University of Tennessee—Knoxville, Knoxville, Tennessee, USA; University of Georgia

**Keywords:** *Campylobacter jejuni*, FlgX, MotA, MotB, chaperone, flagellar motility, stator

## Abstract

The stator units of the flagellum supply power to the flagellar motor via ion transport across the cytoplasmic membrane and generate torque on the rotor for rotation. Flagellar motors across bacterial species have evolved adaptations that impact and enhance stator function to meet the demands of each species, including producing stator units using different fuel types or various stator units for different motility modalities. Campylobacter jejuni produces one of the most complex and powerful flagellar motors by positioning 17 stator units at a greater radial distance than in most other bacteria to increase power and torque for high velocity of motility. We report another evolutionary adaptation impacting flagellar stators by identifying FlgX as a chaperone for C. jejuni stator units to ensure sufficient power and torque for flagellar rotation and motility. We discovered that FlgX maintains MotA and MotB stator protein integrity likely through a direct interaction with MotA that prevents their degradation. Suppressor analysis suggested that the physiology of C. jejuni drives the requirement for FlgX to protect stator units from proteolysis by the FtsH protease complex. C. jejuni Δ*flgX* was strongly attenuated for colonization of the natural avian host, but colonization capacity was greatly restored by a single mutation in MotA. These findings suggest that the likely sole function of FlgX is to preserve stator unit integrity for the motility required for host interactions. Our findings demonstrate another evolved adaptation in motile bacteria to ensure the equipment of the flagellar motor with sufficient power to generate torque for motility.

## Abstract (precis)

The bacterial flagellum is a reversible rotating motor powered by ion transport through stator units, which also exert torque on the rotor component to turn the flagellum for motility. Species-specific adaptations to flagellar motors impact stator function to meet the demands of each species to sufficiently power flagellar rotation. We identified another evolutionary adaptation by discovering that FlgX of Campylobacter jejuni preserves the integrity of stator units by functioning as a chaperone to protect stator proteins from degradation by the FtsH protease complex due to the physiology of the bacterium. FlgX is required to maintain a level of stator units sufficient to power the naturally high-torque flagellar motor of C. jejuni for motility in intestinal mucosal layers to colonize hosts. Our work continues to identify an increasing number of adaptations to flagellar motors across bacterial species that provide the mechanics necessary for producing an effective rotating nanomachine for motility.

## INTRODUCTION

Along with the F_1_F_0_ ATPase and the archaellum, the bacterial flagellum is one of the three known reversible rotary motors produced in nature (1, 2). Flagellar rotation for motility is powered by ion flow through membrane stator units that integrate around the cytoplasmic rotor at the base of a flagellum (3–7). Ion flow through stator units is hypothesized to cause a conformational change resulting in a pushing force that exerts torque upon the rotor to turn the extracellular filament to propel bacteria through different environmental milieus (8).

Components forming the stator units are fairly extensively conserved across motile bacterial species. As studied in Escherichia coli and *Salmonella* species, each stator unit is a heterohexamer of MotA and MotB (MotA_4_MotB_2_) (9, 10). Both proteins are localized to the inner membrane, with MotA possessing four transmembrane domains and two large cytoplasmic domains whereas MotB has one transmembrane domain linked to a large periplasmic domain (11–15). MotB transmembrane domains complete formation of an ion channel upon complexing with MotA proteins to form a stator unit. MotB also contains the conserved aspartate residue for ion binding and translocation (16). Stator unit incorporation into the motor results in conformational changes that remove a “plug” from the channel, allowing ion passage, which in turn results in a major restructuring of the MotB periplasmic domain to interact with peptidoglycan for the tethering that is essential for stator and motor function (17–21). Ion passage also causes conformational changes in MotA within a stator unit such that the cytoplasmic region contacting FliG of the rotor creates a pushing force that generates torque for flagellar rotation (8, 22, 23).

The mechanics of stator function are thought to be largely conserved across flagellar motors of diverse bacterial species. However, some significant differences have been noted. In many *Vibrio* species, motility is powered by sodium ions through the PomAB stator, which is similar to the MotAB counterpart that is powered by hydrogen ions (24–26). Some motile bacteria such as Shewanella oneidensis and Bacillus subtilis possess both hydrogen-powered and sodium-powered stator units, which increases the fuel options for powering motility (27, 28). In pseudomonads, two different stator units, MotAB and MotCD, are produced and exchanged in the motors (29–31). Whereas MotAB stators are used only for swimming motility, MotCD stators are employed for both swimming motility and swarming motility.

E. coli and *Salmonella* stator units are dynamic components of the flagellar motor that can differ in number per motor and can also be exchanged (32–36). Although 1 stator unit can power rotation, up to 11 can be incorporated per motor in a load-dependent manner that correlates with increased torque (32, 34). However, other bacterial species, notably, Campylobacter jejuni, Helicobacter pylori, and *Vibrio* species, produce motors with evolutionary adaptations that appear as disk or ring appendages that impact stator integration and, consequently, the supply of power and generation of torque (3, 37–41). These structures form around the periplasmic rod and rings and radiate outward to serve as scaffolds, resulting in integration of stator units at a greater radial distance around the flagellar rotor than is seen with *Salmonella* (3, 37–39). Due to expanded rotor diameter compared to the *Salmonella* flagellar motor, C. jejuni and *Vibrio* motors accommodate 17 and 13 stator units into the respective flagellar motors (3). The increased number of stator units and the placement of the stator units at a greater radial distance from the axle of the flagellar motor are thought to contribute to increased torque and to the observed higher velocity of motility of the C. jejuni and *Vibrio* motors (3, 37, 42, 43). A current hypothesis is that the number of stator units in flagellar motors with these scaffolds (such as in C. jejuni) is fixed and may not vary depending on the load imparted on the flagellum as seen in E. coli or *Salmonella* (36, 37).

In this work, we expanded the adaptations in flagellar motors that impact stator function and motor output. We determined that C. jejuni FlgX, which was previously identified to be required for motility (44), is a chaperone that functions to maintain stator unit integrity for host colonization and full motility. The use of a chaperone for the stator units is an unusual feature in motile bacteria, as a chaperone specific for stator units has not been recognized in other flagellar systems. We describe additional findings that suggest that the requirement for FlgX-dependent stability of stator units may be due to a specific characteristic of the physiology of C. jejuni, with the FtsH inner membrane protease as a major contributor to stator protein degradation. This work provides insight into the variety of flagellar motor adaptations that have occurred in bacterial species that influence mechanics to ensure that the flagellar motor is equipped with sufficient power and torque for motility.

## RESULTS

### FlgX is required for stator protein integrity.

Gao et al. previously identified FlgX (C. jejuni 81176_0199 [Cjj81176_0199]) as a protein required for wild-type (WT) levels of invasion of Cos-1 cells and flagellar motility (44). Given that FlgX coimmunoprecipitated the MotA and MotB stator proteins in a complex (44), FlgX was proposed to impact flagellar motor assembly or function. However, a specific role for FlgX was not further explored.

**FIG 1:**
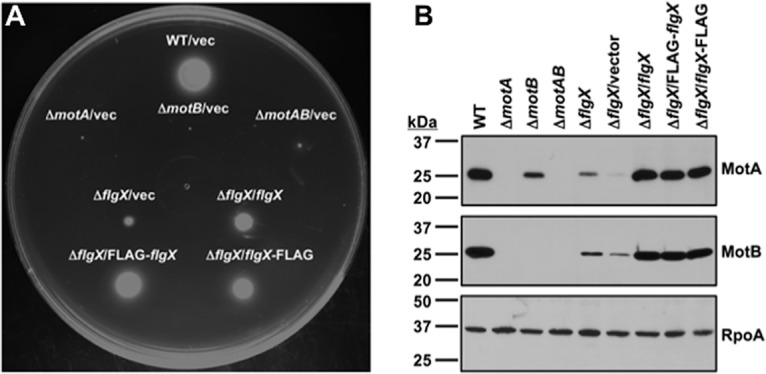
Requirement of FlgX for motility and stability of MotA and MotB stator proteins. For panels A and B, the C. jejuni Δ*flgX* mutant contained a deletion of codons 6 to 153 of the larger predicted coding sequence and all complementing plasmids expressed *flgX* encoding the predicted 165-amino-acid-long protein. (A) Motility phenotypes of WT C. jejuni and isogenic mutants. Motility was analyzed at 30 h of incubation in MH motility agar (0.4% agar) at 37°C under microaerobic conditions. Each strain contained vector alone (vec) or vector used to produce FlgX, FLAG-FlgX, or FlgX-FLAG as indicated. (B) Immunoblot analysis of MotA and MotB stator proteins in whole-cell lysates of WT C. jejuni and isogenic mutants. Specific antiserum to MotA and MotB was used to detect each protein. Detection of RpoA served as a control to ensure equal loading of proteins across strains. C. jejuni Δ*flgX* mutants were complemented with vector alone or plasmid to produce FlgX, FLAG-FlgX, or FlgX-FLAG as indicated.

In order to determine a specific function for FlgX, we created two C. jejuni mutants with different in-frame deletions of the *flgX* coding sequence on the chromosome of C. jejuni strain 81-176 due to uncertainty of the correct start codon for translation. FlgX is predicted to encode a protein of either 165 amino acids or 66 amino acids (the C-terminal 66 amino acids of the longer predicted coding sequence). Neither the shorter nor the longer coding sequence showed similarity to potential orthologs outside *Campylobacter* and other closely related species. On the basis of the longer coding sequence, we generated an in-frame deletion of *flgX* on the chromosome that deleted codons 6 to 153 (DAR2340) and another that deleted codons 101 to 153 (DAR4803). Consistent with the previous study (44), both C. jejuni Δ*flgX* mutants exhibited reduced motility in Mueller-Hinton (MH) motility agar and in liquid broth as visualized by dark-field microscopy even though the mutant populations produced flagella at levels similar to those produced by the WT (Fig. 1A [see also Fig. S1 in the supplemental material] and data not shown). The level of motility was slightly greater than that seen with the Δ*motA* and Δ*motB* mutants, which were completely nonmotile. Complementation with a plasmid to enable constitutive expression of the 66-amino-acid-long version of FlgX did not restore motility to either Δ*flgX* mutant (data not shown). However, complementation with a 165-amino-acid-long version of WT FlgX alone or with an N-terminal or C-terminal FLAG tag restored motility to close to WT levels (Fig. 1A). These findings suggested that the functional FlgX protein is 165 amino acids in length with a predicted size of 19.5 kDa. Unless otherwise indicated, all subsequent studies employed DAR2340 (strain 81-176 *rpsL*^Sm^ Δ*flgX* [where “Sm” represents streptomycin] with a deletion of codons 6 to 153 of *flgX*).

10.1128/mBio.01732-19.1 FIG S1 Comparison of C. jejuni Δ*flgX* mutants for analysis of levels of motility. Motility was analyzed after 30 h of incubation of C. jejuni in MH motility agar (0.4% agar) at 37°C under microaerobic conditions. DAR2340 is C. jejuni 81-176 *rpsL*^Sm^, which contains an in-frame deletion of codons 6 to 153 of *flgX*, whereas DAR4803 contains a smaller in-frame deletion of only codons 101 to 153 of *flgX*. Download FIG S1, TIF file, 1.1 MB. Copyright © 2019 Ribardo et al. 2019 Ribardo et al. This content is distributed under the terms of the Creative Commons Attribution 4.0 International license.

Analysis of stator proteins in WT C. jejuni and the Δ*flgX* mutant revealed that FlgX is required for production of WT levels of both MotA and MotB (Fig. 1B). MotA and MotB are encoded in an operon with *motA* directly upstream of *motB*. As observed in other motile bacteria, C. jejuni MotA and MotB are dependent upon each other for full stability (Fig. 1B; see also Fig. S2) (10). Whereas deletion of *motB* caused a reduction in MotA, MotB was barely detectable in the Δ*motA* mutant (Fig. 1B; see also Fig. S2). WT levels of MotA and MotB were restored by in *trans* complementation with the respective gene deleted in each mutant (Fig. S2). Complementation of the Δ*flgX* mutant with a plasmid that constitutively expressed WT FlgX, FLAG-FlgX, or FlgX-FLAG restored WT levels of both stator proteins (Fig. 1B). Note that all FLAG-tagged FlgX proteins used in this experiment and those described were the full-length 165-amino-acid-long sequence. We were unable to compare the relative levels of FlgX produced from the native chromosomal locus with FlgX expressed from complementing plasmids as we were unable to generate specific antiserum against FlgX after multiple attempts. We eliminated the possibility of a role for FlgX in transcription of *motAB*, as semiquantitative real-time PCR (qRT-PCR) analysis revealed similar levels of *motA* and *motB* expression in WT C. jejuni and the Δ*flgX* mutant with or without a complementing plasmid for expression of FlgX (Fig. S3). Thus, FlgX functions posttranslationally to support WT levels of MotA and MotB stator components.

10.1128/mBio.01732-19.2 FIG S2 Interdependency of MotA and MotB for stability. Immunoblots of MotA and MotB stator protein levels in whole-cell lysates of WT C. jejuni and isogenic mutants are shown. Specific antiserum to MotA and MotB was used to detect each protein. Detection of RpoA served as a control to ensure equal levels of loading of proteins across strains. C. jejuni Δ*motA* or Δ*motB* mutants were complemented with vector alone or vector expressing *motA* or *motB*. Download FIG S2, TIF file, 1.1 MB. Copyright © 2019 Ribardo et al. 2019 Ribardo et al. This content is distributed under the terms of the Creative Commons Attribution 4.0 International license.

10.1128/mBio.01732-19.3 FIG S3 Transcriptional analysis of C. jejuni *motA* and *motB*. Semiquantitative real-time PCR analysis of transcription of *motA* and *motB* in WT C. jejuni containing vector alone and C. jejuni Δ*flgX* containing either vector alone or a vector to express *flgX* was performed. The value representing the level of expression of both *motA* and *motB* in WT C. jejuni as measured by qRT-PCR was set to 1, with the level of expression of genes in C. jejuni Δ*flgX* strains shown relative to that of the WT strain. Error bars indicate standard errors. Download FIG S3, TIF file, 1.1 MB. Copyright © 2019 Ribardo et al. 2019 Ribardo et al. This content is distributed under the terms of the Creative Commons Attribution 4.0 International license.

### FlgX interactions with the stator units likely occur through MotA.

**FIG 2:**
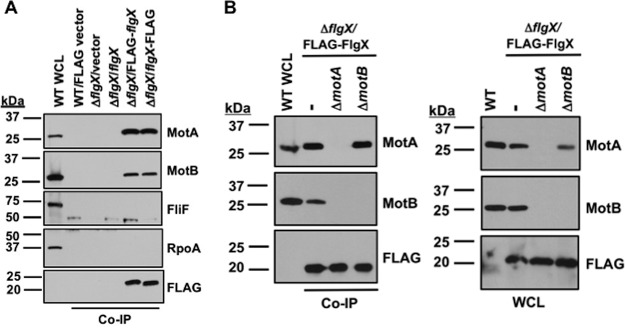
Detection of *in vivo* interactions between FlgX and MotA or MotB in C. jejuni. (A) Analysis of FlgX coimmunoprecipitated (Co-IP) C. jejuni proteins. WT C. jejuni with vector alone and C. jejuni Δ*flgX* complemented with vector alone or vector to produce untagged WT FlgX, FLAG-FlgX, or FlgX-FLAG were included for analysis. Briefly, C. jejuni cells were cross-linked with formaldehyde to trap complexes and then quenched to stop the cross-linking reaction. Cell were pelleted, osmotically lysed, and then solubilized with a solution containing Triton X-100. After centrifugation, FlgX was immunoprecipitated overnight with anti-FLAG M2 affinity gel resin (see Materials and Methods for full details). MotA, MotB, FliF, and RpoA were detected by specific antisera after immunoprecipitation. FLAG-tagged FlgX was detected by a monoclonal anti-FLAG antibody. The first lane contains proteins from whole-cell lysates of WT C. jejuni for reference. Note that, although the FLAG-FlgX and FlgX-FLAG proteins are predicted to be the same size, a difference in the levels of mobility during SDS-PAGE is responsible for the apparent size shift. This possible structural alteration does not appear to impact function as both proteins coimmunoprecipitate with the stator proteins. (B) Analysis of FlgX coimmunoprecipitated proteins in C. jejuni *motA* and *motB* mutants. C. jejuni Δ*flgX*, Δ*flgX* Δ*motA*, or Δ*flgX* Δ*motB* contained a vector to express FLAG-FlgX. MotA and MotB were detected by specific antisera whereas FLAG-FlgX was detected by a monoclonal anti-FLAG antibody after immunoprecipitation with FLAG-FlgX (left) or in whole-cell lysates of the strains analyzed (right).

Gao et al. previously observed interactions of FlgX with MotA and MotB in C. jejuni via coimmunoprecipitation assays (44). We confirmed that MotA and MotB coimmunoprecipitated with both FLAG-FlgX or FlgX-FLAG when expressed in C. jejuni Δ*flgX* after formaldehyde cross-linking of cells (Fig. 2A). MotA and MotB did not bind the immunoprecipitation resin nonspecifically as these proteins were not detected in samples in which WT FlgX without a FLAG tag was produced or in a sample lacking FlgX (Fig. 2A). This interaction between FlgX and the stator proteins was specific as FlgX did not coimmunoprecipitate the FliF MS ring protein, which is an inner membrane flagellar protein like the stator proteins, or the cytoplasmic α subunit of RNA polymerase (RpoA; Fig. 2A).

We next conducted experiments to discern whether FlgX interacted with MotA or with MotB or with both proteins to maintain stator protein stability and function. For this analysis, we constructed mutants lacking either *motA* or *motB* in C. jejuni Δ*flgX* and then expressed FLAG-FlgX in *trans* from a plasmid for coimmunoprecipitation assays. Native MotA immunoprecipitated with FLAG-FlgX at similar levels in the Δ*flgX* and Δ*flgX* Δ*motB* mutants, even though the MotA levels were slightly lower in the Δ*flgX* Δ*motB* mutant (Fig. 2B). These data indicated that FlgX-MotA interactions do not require MotB. Due to significantly reduced levels of MotB in the Δ*motA* mutant (Fig. 2B), we were unable to determine whether FlgX also directly interacts with MotB.

**FIG 3:**
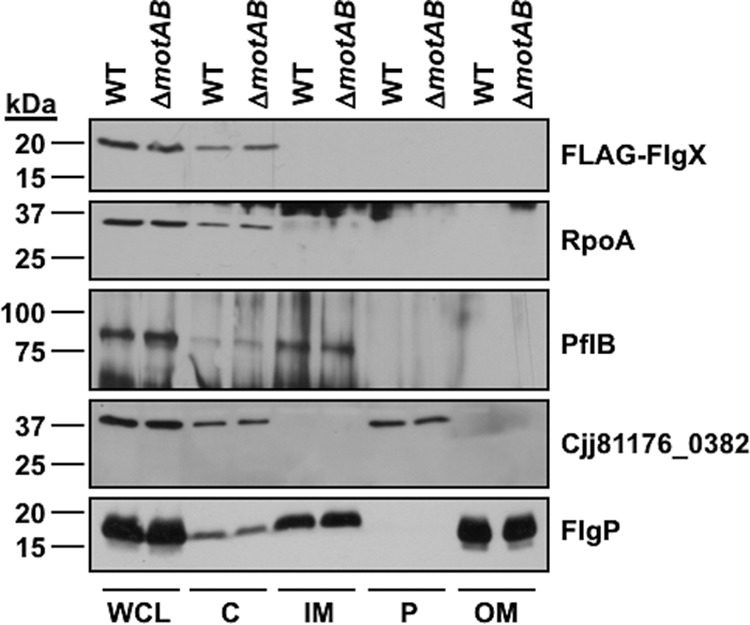
Localization of FlgX in C. jejuni. Data represent results of immunoblot analysis of FLAG-FlgX localization in different cellular fractions of WT C. jejuni or C. jejuni Δ*motAB*. C. jejuni cultures were standardized to similar cellular densities and then fractionated to recover proteins from the cytoplasmic (C), inner membrane (IM), periplasmic (P), and outer membrane (OM) fractions. Samples from whole-cell lysates (WCL) were also recovered. Equal amounts of proteins were loaded for all strains. FLAG-FlgX was detected by a monoclonal anti-FLAG antibody. Specific antisera were used to detect proteins as specific markers for different fractions, including RpoA (cytoplasm), PflB (inner membrane), Cjj81176_0382 (periplasm), and FlgP (outer membrane).

FlgX is predicted to be a cytoplasmic protein, lacking both a signal sequence for secretion and transmembrane domains for integration into the inner membrane. We isolated proteins from the cytoplasmic, membrane, and periplasmic fractions of C. jejuni Δ*flgX* and Δ*flgX* Δ*motAB* mutants producing FLAG-FlgX to determine the compartmental location of FlgX in C. jejuni. As predicted, we observed FlgX to localize exclusively to the C. jejuni cytoplasm with RpoA and not with any other compartment (Fig. 3). Although we anticipated that FlgX might show some localization to the inner membrane due to interactions with stator units, FlgX was not found to be associated with the inner membrane (Fig. 3). Thus, the cytoplasmic location did not change regardless of whether the stator proteins were produced. We propose that FlgX interacts with a cytoplasmic domain of MotA to maintain stability of MotAB stator units in C. jejuni on the basis of the following observations: (i) FlgX is localized to the cytoplasm; (ii) MotA has prominent cytoplasmic domains, in contrast to MotB, which is predicted to have a small N-terminal cytoplasmic domain of only ∼18 residues; and (iii) FlgX efficiently coimmunoprecipitated MotA in the absence of MotB.

**FIG 4:**
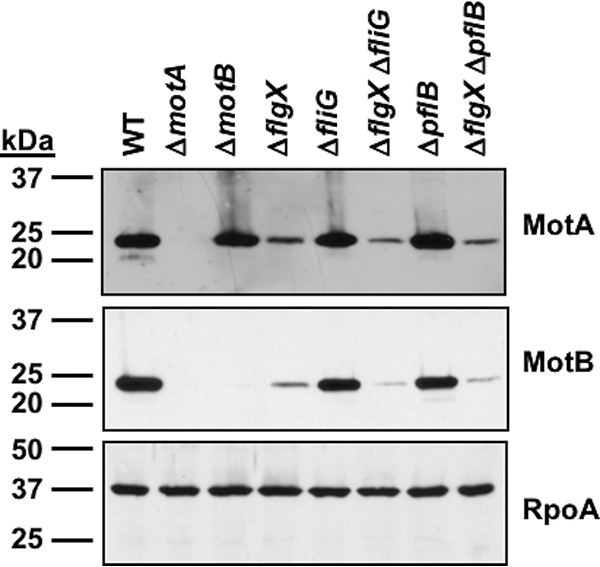
FlgX-dependent stability of MotA and MotB stator proteins in flagellated amotile mutants. Data represent results of immunoblot analysis of MotA and MotB stator proteins in whole-cell lysates of WT C. jejuni and isogenic single or double mutants that result in flagellated but amotile mutants. Specific antiserum to MotA and MotB was used to detect each protein. Detection of RpoA served as a control to ensure equal loading of proteins across strains.

Although stator units are dynamic components of the *Salmonella* and E. coli flagellar motors, a current hypothesis suggests that the stator units of C. jejuni and other bacteria that integrate stators into the flagellar motor via scaffolding proteins are fixed and may not fluctuate in number with the load exerted upon the flagellum (37). Previous structural analysis of the C. jejuni flagellar motor by electron cryotomography revealed that 17 stator complexes are integrated into the flagellar motor, with MotA contacting FliG and MotB (likely interacting with the PflB proximal disk scaffolding protein) (3). In line with these observations, C. jejuni Δ*pflB* lacks MotAB stator units in the flagellar motor (3). We determined whether FlgX is required for stability of stator proteins in a nonfunctional motor without a rotor (Δ*fliG* mutant) or when stator units are unable to integrate into the flagellar motor (Δ*pflB* mutant). We observed that FlgX was required to maintain WT levels of the stator proteins in the flagellar motors of both the Δ*fliG* mutant and the Δ*pflB* mutant (Fig. 4). Due to uncertainty with respect to analyzing C. jejuni under conditions in which all stator units would be integrated around the rotor without any unincorporated stator units produced elsewhere in the cell, we were unable to determine whether FlgX remains associated with stator units after their incorporation into the motor to maintain their integrity.

### Suppressor analysis of C. jejuni Δ*flgX*.

**FIG 5:**
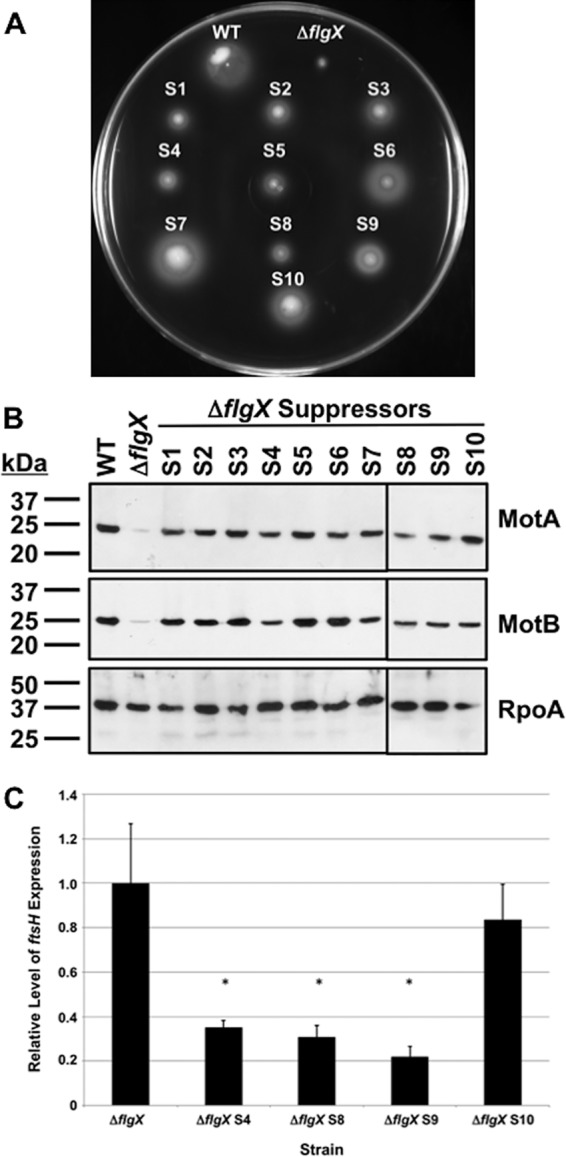
Analysis of C. jejuni Δ*flgX* suppressor mutants with restored motility. (A to C) DAR4803, representing strain 81-176 *rpsL*^Sm^ Δ*flgX* with deletion of codons 101 to 153 of *flgX*, and derived isogenic suppressor mutants (annotated as “S1” through “S10”) are shown. (A) Motility phenotypes of WT C. jejuni, the parental Δ*flgX* mutant, and isolated motile Δ*flgX* suppressor mutants. Motility was analyzed after 30 h of incubation in MH motility agar (0.4% agar) at 37**°**C under microaerobic conditions. (B) Immunoblot analysis of MotA and MotB stator protein levels in whole-cell lysates of WT C. jejuni, the parental Δ*flgX* mutant, and isolated motile Δ*flgX* suppressor mutants. Specific antiserum to MotA and MotB was used to detect each protein. Detection of RpoA served as a control to ensure equal loading of proteins across strains. (C) Semiquantitative real-time PCR analysis of transcription of *ftsH* in the parental Δ*flgX* mutant and selected motile Δ*flgX* suppressor mutants. Suppressor mutants S4, S8, and S9 contain frameshift mutations in *Cjj81176_1135*. Suppressor mutant S10 with the *motA*_H138Y_ missense mutation served as a control. The level of expression of *ftsH* in C. jejuni Δ*flgX* as measured by qRT-PCR is set to 1. Expression of *ftsH* suppressor mutants is shown relative to that seen with C. jejuni Δ*flgX*. Error bars indicate standard deviations. Statistically significant differences in *ftsH* expression levels between C. jejuni Δ*flgX* and suppressor mutants (*, *P* < 0.05) as performed by Student's *t* test are indicated.

Taking into consideration the results presented above, we propose that FlgX likely functions as a chaperone to preserve the integrity of C. jejuni stator units to ensure power and generation of torque for flagellar motility. We performed a suppressor analysis with C. jejuni Δ*flgX* to attempt to isolate mutants with augmented motility and to provide insight into how FlgX ensures stator integrity as a chaperone. For this analysis, we inoculated C. jejuni Δ*flgX* mutants (with an in-frame deletion of residues 6 to 153 or residues 101 to 153) into MH motility agar, followed by incubation at 37°C for up to 7 days. A total of 18 independent suppressor mutants were isolated from motile flares originating from the point of inoculation. All Δ*flgX* suppressor mutants demonstrated a greater level of motility and higher levels of MotA and MotB in lysates than the parental Δ*flgX* strains. Panels A and B of Fig. 5 show the motility phenotypes and stator protein levels for all Δ*flgX* suppressor mutants isolated from the mutant with a deletion of codons 101 to 153 of *flgX* (suppressors S1 to S10). Similar elevated levels of motility and MotA and MotB stator proteins were also observed for the eight suppressor mutants isolated from the C. jejuni Δ*flgX* lacking codons 6 to 153 (suppressors S11 to S18; data not shown).

Genomic sequencing or targeted sequencing of genes of interest in the Δ*flgX* suppressor mutants revealed three classes of suppressor mutations (Table 1). The first class of suppressor mutations involved five alterations to the *motAB* locus. Four suppressor mutations were identified as point mutations in *motA* to produce MotA_H138Y_ (3 independent isolates) and MotA_A201V_ (a single isolate). The H138 residue is located in the larger of the two cytoplasmic domains of MotA (Fig. S4A and B). This domain interacts with FliG for torque generation and rotor rotation, but H138Y is not hypothesized to be involved in these processes. The A201 residue is predicted to be the last residue of the most C-terminal transmembrane domain of MotA (Fig. S4A and B). The suppressor mutation in suppressor S7 resulted in a duplication of a 13-kb region of the chromosome encompassing *motAB*, effectively creating a *motAB* merodiploid that increased MotA and MotB production in the absence of FlgX (Fig. 5B).

**TABLE 1:** Identification of C. jejuni Δ*flgX* suppressor mutations

Suppressor mutant^*a*^	Gene^*b*^	Mutation	Effect
S1	*ftsH*	G750T transversion	FtsH_M250I_
S2	*ftsH*	Duplication of bases 393–410	FtsH S131-V136 repeated
S3	*ftsH*	G750T transversion	FtsH_M250I_
S4	*Cjj81176_1135*	Insertion of AA at base 282	Frameshift
S5	*ftsH*	G750T transversion	FtsH_M250I_
S6	*ftsH*	Duplication of bases 87–296	FtsH_K98N_ with F29-K98 repeated
S7	*motAB* region	Duplication of 13-kb region	*motAB* merodiploid
S8	*Cjj81176_1135*	Insertion of A at base 12	Frameshift
S9	*Cjj81176_1135*	Deletion of bases 42–54	Frameshift
S10	*motA*	C414T transition	MotA_H138Y_
S11	*motA*	C414T transition	MotA_H138Y_
S12	*Cjj81176_1135*	Insertion of T at base 231	Frameshift
S13	*Cjj81176_1135*	Deletion of A at base 12	Frameshift
S14	*motA*	A601G transition	MotA_A201V_
S15	*ftsH*	T272A transversion	FtsH_S243T_
S16	*ftsH*	G750T transversion	FtsH_M250I_
S17	*motA*	C414T transition	MotA_H138Y_
S18	*Cjj81176_1135*	Insertion of A at base 12	Frameshift

a Suppressor mutants S1 to S10 were isolated from DAR4803 (containing a deletion of codons 101 to 153 of *flgX*), and mutants S11 to S18 were isolated from DAR2340 (containing a deletion of codons 6 to 153 of *flgX*). Spontaneous motility mutants were isolated from motility agar after incubation at 37°C for up to 8 days.

b The location of the mutation was identified by genomic sequencing or by sequencing of a specific gene of interest.

10.1128/mBio.01732-19.4 FIG S4 Location of MotA suppressor mutations. (A) Predicted topology of C. jejuni MotA with important residues predicted for MotA function and residues identified by suppressor analysis that restored motility to C. jejuni Δ*flgX*. Transmembrane domains that insert in the inner membrane were predicted by the use of TMpred software. Residues with transmembrane domains are shown with the boundaries of each transmembrane region indicated. The H138 and A201 residues that were mutated in suppressor mutants that restored motility to C. jejuni Δ*flgX* are indicated in red. The large cytoplasmic domain of MotA that contains the R89 and E97 residues (in blue) predicted to interact with specific residues in the C-terminal domain (CTD) of FliG (in black) to generate torque and rotor rotation is shown. (B) Clustal Omega sequence alignments of Salmonella enterica serovar Typhimurium strain 14028S MotA (St_MotA), C. jejuni 81-176 MotA (Cj_MotA), and H. pylori G27 MotA (Hp_MotA) are shown. Gray regions show predicted transmembrane domains from each protein with black bars labeled “TM1” through “TM4” demarcating the transmembrane domains of *Salmonella* MotA. Blue residues indicate the predicted or known residues of the MotA proteins that interact with FliG to generate torque and rotor rotation. The residues of WT C. jejuni MotA that were mutated to H138Y and A201V in the isolated C. jejuni Δ*flgX* suppressor mutants that were restored for motility are shown. Note that H. pylori MotA naturally has a tyrosine in place of H138. Download FIG S4, TIF file, 1.9 MB. Copyright © 2019 Ribardo et al. 2019 Ribardo et al. This content is distributed under the terms of the Creative Commons Attribution 4.0 International license.

The members of the second class of suppressor mutants all contained mutations within *ftsH*, which encodes an essential inner membrane AAA protease that largely assists in quality control of inner membrane proteins, along with degradation of some cytoplasmic proteins (Table 1) (45). Four Δ*flgX* suppressor mutants contained independent transversions occurring at the same nucleotide to result in production of FtsH_M250I_, and another suppressor mutation was due to a transversion to produce FtsH_S243T_. In structurally analyzed FtsH proteins, these residues are near the entry pore of the FtsH ATPase complex and are possibly important for pore substrate recognition of misfolded substrate proteins (46). Two other suppressor mutations identified were in-frame duplications of part of the coding region for *ftsH*, including an 18-nucleotide duplication and a 210-nucleotide duplication (Table 1).

The members of the final class of suppressor mutants all contained alterations within *Cjj81176_1135*, encoding a predicted PrmA homolog that functions as the L11 ribosomal protein methyltransferase (Table 1). *Cjj81176_1135* is immediately upstream of *ftsH*, and the two genes are likely cotranscribed. All six mutations in *Cjj81176_1135* involved insertions or deletions of nucleotides that caused frameshift mutations and truncation of the *Cjj81176_1135* reading frame. We hypothesized that these frameshift mutations in *Cjj81176_1135* may have caused polar effects on transcription or the on stability of the *ftsH* mRNA. qRT-PCR analysis revealed that the *ftsH* mRNA levels in these *Cjj81176_1135* mutants were 3-fold to 5-fold lower than in the parental Δ*flgX* mutant or in a Δ*flgX motA*_H138Y_ suppressor (suppressor S10) that was also identified in this suppressor screen (Fig. 5C). Thus, a reduction in the level of *ftsH* transcription in these *Cjj81176_1135* mutants likely resulted in the increased levels of MotA and MotB in the absence of FlgX.

**FIG 6:**
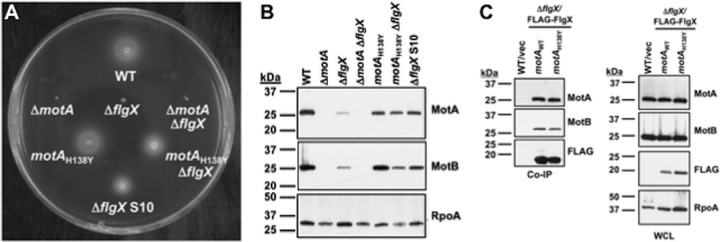
Sufficiency of MotA_H138Y_ to suppress C. jejuni Δ*flgX* phenotypes. (A) Motility phenotypes of WT C. jejuni and isogenic mutants. Motility was analyzed after 30 h of incubation in MH motility agar (0.4% agar) at 37**°**C under microaerobic conditions. (B) Immunoblot analysis of MotA and MotB stator protein levels in whole-cell lysates of WT C. jejuni and isogenic mutants. Specific antiserum to MotA and MotB was used to detect each protein. Detection of RpoA served as a control to ensure equal loading of proteins across strains. In panels A and B, C. jejuni strains contained WT *motA* or an in-frame deletion of *motA* (Δ*motA*) or the WT *motA* gene was replaced on the chromosome with *motA*_H138Y_. (C) Analysis of FlgX coimmunoprecipitation of WT MotA and MotA_H138Y_ in C. jejuni. C. jejuni Δ*flgX* and Δ*flgX motA*_H138Y_ contained a vector to express FLAG-FlgX. MotA and MotB were detected by specific antisera, whereas FLAG-FlgX was detected by a monoclonal anti-FLAG antibody in proteins after immunoprecipitation with FLAG-FlgX (left) or in whole-cell lysates of the strains analyzed (right).

We examined the sufficiency of the H138Y alteration of MotA for restoring stator protein levels and motility to the Δ*flgX* mutant by replacing WT *motA* gene with *motA*_H138Y_ on the chromosome of WT C. jejuni 81-176 and the Δ*flgX* mutant. MotA topology predictions suggest that H138Y resides in the first of two large cytoplasmic domains of the protein. The levels of motility, in addition to those of both MotA and MotB proteins, were comparable between WT C. jejuni and C. jejuni *motA_H138Y_* (Fig. 6A and B). Importantly, MotA and MotB levels were increased in the Δ*flgX motA_H138Y_* mutant compared to the Δ*flgX* mutant, and these levels were similar to those of the original Δ*flgX motA*_H138Y_ suppressor mutant (S10) that had been isolated. Furthermore, we found that the Δ*flgX motA_H138Y_* mutant supported a greater level of motility than the Δ*flgX* mutant containing WT *motA* (Fig. 6A). Measurements of the motile rings in motility agar indicated that the level of migration from the point of inoculation of the Δ*flgX motA_H138Y_* mutant was 83.5% (±19.5%, *P* < 0.05) of the level seen with WT C. jejuni, indicating only a modest but a significant reduction in motility. These observations demonstrate that the H138Y alteration in MotA was nearly sufficient to restore the levels of integrity and function of the stator units seen in the absence of FlgX to WT levels. We also observed that H138 was not essential for binding by FlgX, as FLAG-FlgX coimmunoprecipitated WT MotA and MotA_H138Y_ comparably well (Fig. 6C).

We attempted to determine whether the FtsH_M250I_ mutation was sufficient for restoring stator protein levels and motility to the Δ*flgX* mutant. However, we were unable to replace WT FtsH with FtsH_M250I_ because construction of such a mutant requires an intermediate step to disrupt wild-type *ftsH* (an essential gene) with an antibiotic resistance cassette prior to replacement with the *ftsH*_M250I_ allele. We also attempted to construct plasmids to express WT *ftsH* and *ftsH*_M250I_ in *trans* and then to eliminate *ftsH* on the chromosome. However, the level of in *trans* expression of either allele from multicopy plasmids was likely too high relative to native *ftsH* expression from the chromosome to accurately interpret whether FtsH_M250I_ had reduced protease activity with respect to the stator proteins (data not shown).

### Analysis of stator proteins upon heterologous expression in C. jejuni and E. coli.

As we are unaware of the existence of any previous report identifying a chaperone specific for stator units in a bacterial flagellar system, we investigated whether the requirement of FlgX as a chaperone for stator units is linked to an aspect of C. jejuni physiology and/or to a specific feature of the C. jejuni stator proteins. For this approach, we compared the levels of production of C. jejuni and Salmonella enterica serovar Typhimurium MotAB stator units upon heterologous expression in C. jejuni Δ*motAB* in the presence or absence of FlgX and in E. coli. We complemented the C. jejuni and E. coli strains with plasmids to express *Salmonella motAB* or C. jejuni *motAB* from identical promoters and plasmid backbones to ensure levels of protein production that were as similar as possible. FLAG-tagged epitopes were fused to the C terminus of MotB for each *motAB* locus so that the same antibody could be used to detect MotB-FLAG to eliminate differences in methods for protein detection. We attempted to fuse a FLAG tag to the N terminus of MotA for similar analyses, but FLAG-MotA does not efficiently complement C. jejuni Δ*motA* and the protein was not stable for detection.

**FIG 7:**
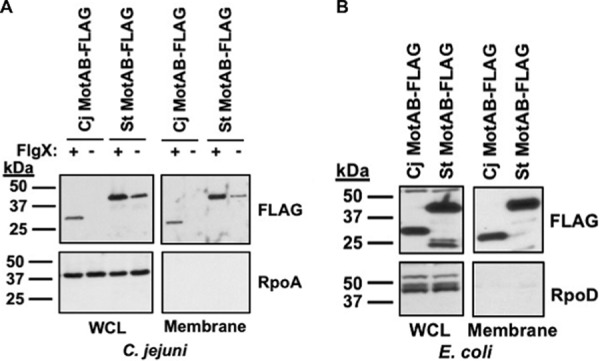
Stability of stator units upon heterologous expression. For panels A and B, the coding sequences of the *motAB* loci from C. jejuni and S. enterica serovar Typhimurium were expressed from the same constitutive promoter and plasmid backbone in C. jejuni strain Δ*motAB* with production of WT FlgX (+) or without production of FlgX (−) (A) or in E. coli (B). A FLAG tag was fused to the C terminus of the MotB proteins to monitor stator production with the same anti-FLAG antibody in whole-cell lysates (WCL) or in the total membrane fraction. Detection of C. jejuni RpoA or E. coli RpoD served as a control to ensure equal loading of proteins across strains and as a control for fractionation.

Expression of C. jejuni *motAB-FLAG* and *Salmonella motAB-FLAG* in C. jejuni Δ*motAB* containing FlgX revealed that both MotB-FLAG proteins (and presumably MotA partner proteins) were stable, with an apparent increased level of *Salmonella* MotB present compared to C. jejuni MotB (Fig. 7A). The differences in the sizes of the MotB proteins was due to *Salmonella motB* naturally encoding a larger protein. Whereas no C. jejuni MotB-FLAG was detected in C. jejuni Δ*motAB* without FlgX, *Salmonella* MotB-FLAG was detected in both the lysates and the total membrane fraction in this strain. These data suggest that the *Salmonella* stator unit is not as sensitive to degradation under the physiological conditions present in C. jejuni as the native C. jejuni stator proteins. We also noticed higher levels of *Salmonella* MotB-FLAG in C. jejuni with FlgX than without, indicating that FlgX has an ability to enhance levels of heterologous stator proteins. In contrast to expression in C. jejuni, the C. jejuni stator proteins and the *Salmonella* stator proteins were produced at comparable levels and were stable in E. coli, which does not encode any discernible FlgX ortholog (Fig. 7B). Therefore, C. jejuni stators are stable without FlgX in other bacterial species. These observations point to the physiology of C. jejuni, presumably due to its altered or augmented FtsH activity, targeting C. jejuni stator proteins for degradation and thus requiring FlgX as a chaperone for maintaining stator unit integrity for flagellar motor rotation and motility.

### *In vivo* requirement of FlgX for host colonization.

**FIG 8:**
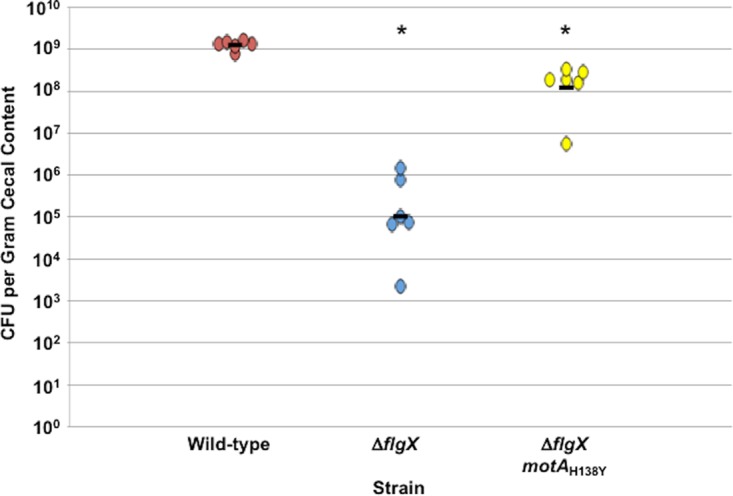
Commensal colonization capacity of WT C. jejuni and Δ*flgX* and and Δ*flgX motA*_H138Y_ suppressor mutants in avian hosts. Day-of-hatch chicks were orally gavaged with WT C. jejuni 81-176 Sm^r^ (red), the Δ*flgX* mutant (blue), or the reconstructed Δ*flgX motA*_H138Y_ suppressor mutant (yellow). The measured levels of inocula for the chicks as determined by dilution plating were as follows: 2.1 × 10^4^ CFU for WT C. jejuni; 2.06 × 10^4^ CFU for theΔ*flgX* mutant; and 9.7 × 10^3^ CFU for the Δ*flgX motA*_H138Y_ mutant. Chicks were sacrificed at day 14 postinfection, and the levels of all C. jejuni strains in the ceca (reported as CFU per gram of content) were determined. Each closed circle represents the level of C. jejuni in a single chick. The black horizontal bars represent the geometric mean for each group. Statistical analysis was performed using the Mann-Whitney *U* test. The asterisks (*) indicate a statistically lower level of colonization of mutants than of WT C. jejuni (*P < *0.05).

C. jejuni requires flagellar motility for infection of humans to promote diarrheal disease and optimal colonization of the intestinal tract of avian species for commensalism (47–50). As stator units are needed to supply power and generate torque for flagellar rotation and motility, we assessed the requirement for FlgX for cecal colonization of the chick intestinal tract. We also examined whether MotA_H138Y_, which promotes increased stator unit stability and motility in the absence of FlgX, could suppress any potential requirement for FlgX for host colonization. We subjected day-of-hatch chicks to orally gavage with ∼10^4^ CFU of WT C. jejuni, the Δ*flgX* mutant, and the reconstructed Δ*flgX motA*_H138Y_ mutant and then determined the level of colonization in the ceca of chicks 14 days postinfection. WT C. jejuni reached an average of 1.24 × 10^9^ CFU per gram of cecal content, but the Δ*flgX* mutant showed a 10,000-fold reduction in colonization (average of 1.04 × 10^5^ CFU per gram of cecal content; Fig. 8). We did not identify any Δ*flgX* suppressor mutants that developed *in vivo* to enhance motility. Replacement of wild-type *motA* with *motA*_H138Y_ in the Δ*flgX* mutant caused the colonization capacity of the Δ*flgX* mutant to improve by 3 orders of magnitude to 1.2 × 10^8^ CFU per gram cecal content on average, which was only 10-fold lower than the level seen with WT C. jejuni. Thus, the colonization defect of the Δ*flgX* mutant was almost completely restored by a single-point mutation in MotA. These data suggest that the primary *in vivo* role of FlgX is as a chaperone to maintain stator stability to supply power and generate torque for flagellar rotation for the motility that is essential for host interactions.

## DISCUSSION

Stators are essential components of the flagellar motor that power rotation by transporting hydrogen or sodium ions and exert torque on the flagellar rotor for rotation. Efficient production and integration of stator units into the flagellar motor are essential for motility. C. jejuni FlgX was initially found to be required for motility and to interact with MotA and MotB (44). In this work, we provide a detailed analysis implicating FlgX as a chaperone that ensures the integrity of the MotAB stator unit for supplying power and torque for flagellar motility. We base this conclusion on the following observations: (i) production of WT levels of MotA and MotB in C. jejuni requires FlgX; (ii) FlgX is not a regulatory factor required for transcription of *motAB*; (iii) FlgX can be isolated in a complex with MotA and MotB, likely through a direct interaction between FlgX and MotA; (iv) FlgX is required for MotA and MotB stability in flagellar mutants unable to integrate stator units into the motor, suggesting that FlgX is required for the stability of stator units prior to incorporation into the motor; and (iv) MotA and MotB levels and motility were partially to fully restored in the Δ*flgX* mutant with suppressor mutations that reduced *ftsH* expression or (presumably) activity of the FtsH inner membrane protease, indicating that FlgX protects stator units from proteolysis. The one issue concerning a function often attributed to some other chaperone proteins that we currently cannot resolve is whether FlgX is released from the stator unit upon integration of the stators into the motor. Unfortunately, we cannot design an experiment that would give strong conclusive data for assessment. To date, a chaperone for stator complexes has not been identified in any other flagellar system. Along with the scaffolding structures composed of FlgP, PflA, and PflB in C. jejuni (3), FlgX represents another unique evolutionary adaptation that has occurred in *Campylobacter* species to influence the mechanics of a high-torque motor for motility.

Our data suggest that FlgX may interact with only MotA to maintain stability of the stator complexes. MotA is predicted to contain two large cytoplasmic domains, whereas MotB is predicted to contain a small N-terminal domain of about 18 amino acids. Because FlgX was found only in the cytoplasm of C. jejuni and most of the cytoplasmic portions of the MotAB stator are composed of MotA domains, it is likely that FlgX interaction with stator units occurs via MotA cytoplasmic domains. Most convincing was our observation that MotA coimmunoprecipitated with FlgX in C. jejuni lacking MotB, demonstrating that FlgX-MotA interactions occur independently of MotB. Since MotA is required for full stability of MotB, FlgX likely promotes MotB stability indirectly by interacting with and maintaining the stability of MotA. If this is true, then our observations of the increased stability of the heterologous *Salmonella* MotB stator component in C. jejuni expressing *Salmonella motAB* and producing FlgX suggest that FlgX may target a common structural epitope in the cytoplasmic domains of MotA proteins for interactions.

Although FlgX was able to increase *Salmonella* MotB (and presumably MotA) stability in C. jejuni, proteins with significant homology to FlgX are present only in *Campylobacter* species and closely related epsilonproteobacterial species. Even in Helicobacter pylori, another epsilonproteobacterium, the idea of the presence of a functional FlgX ortholog is dubious, with only one protein identified with low homology (i.e., HPG27_1307; 28% identical and 36% similar to FlgX in 47 of 183 residues). Considering the number of flagellar systems that have been investigated across bacterial species, C. jejuni and closely related species may represent an exclusive example in producing flagellar systems requiring a protein such as FlgX to function as a chaperone for stator units. This proposition prompts an intriguing question. Why does C. jejuni need FlgX as a chaperone to maintain stator stability when other motile bacterial species apparently do not? On the basis of the results from our suppressor screen, suppressor mutations in *motA* or those in the *ftsH* locus that either reduce the levels of *ftsH* expression or presumably alter FtsH activity allow higher levels of stator proteins and motility in the absence of FlgX. These findings suggest that the physiology of C. jejuni may include an altered or augmented form of FtsH activity relative to other motile bacteria that consequently targets stator proteins for destruction. Currently, we do not know whether the C. jejuni stator proteins are initially produced in C. jejuni in a relatively more unstructured form that might make them more susceptible to degradation by FtsH. However, when C. jejuni stator proteins were produced in E. coli, they were comparable to *Salmonella* stator proteins in stability. This finding suggests the C. jejuni proteins are not generally less structurally stable than other bacterial stator proteins, at least when produced in other systems.

It is unclear why the physiology of C. jejuni might have evolved to include FtsH activity targeting stator units, and perhaps other inner membrane proteins in general, more readily than that in other bacterial species. C. jejuni does have an optimal growth temperature of 42°C, which is the normal body temperature of its natural avian host. It is possible that this optimal growth temperature may have necessitated an elevated or altered form of FtsH activity as a quality control mechanism for increased protein unfolding and turnover. However, altered FtsH activity might be expected to target many proteins nonspecifically such that numerous proteins, including the MotA and MotB stator proteins, would require their own specific chaperones, which has not been a common theme revealed in C. jejuni biology so far.

Another intriguing possibility is that targeted destruction of the stator units by FtsH in C. jejuni might be a mechanism to remove incorporated stators from the flagellar motor to reduce power and rotation. Currently, it is unknown whether stator units in the flagellum are dynamic or whether the stator number is directly correlated to the load exerted on the flagellum in C. jejuni. In E. coli, one stator complex is sufficient to power rotation, but stator numbers increase to up to 11 per motor to augment rotational speed and power when the external load exerted on the flagellum rises (33–36, 51, 52). A current hypothesis suggests that the stator number in the C. jejuni flagellar motor may be fixed at 17 stator units per motor (3, 37). If FlgX is released from stator units after incorporation into the motor, targeting these incorporated stator units for proteolysis by FtsH may be a mechanism to reduce power for rotation and to disassemble stator units from the flagellar motor. Currently, we are unable to discern whether FlgX is associated with stators after incorporation into the motor. However, it is difficult to envision the existence of a natural niche or condition in which C. jejuni would need to reduce stator function and torque since the bacterium is found primarily associated with intestinal mucus layers in avian, animal, or human hosts. Production of a high-torque flagellar motor seems optimal for motility through viscous milieus such as these host mucus layers. Thus, evolving FlgX as an adaptation to the flagellar motor of C. jejuni likely ensures optimal production of stator units to fully and consistently supply power and torque for the high-torque flagellar motor.

We also isolated suppressor mutations within *motA* that partially restored motility, with *motA*_H138Y_ almost completely restoring motility to the Δ*flgX* mutant. The H138 residue of MotA is not required for interactions with FlgX as the mutant protein appeared to coimmunoprecipitate with FlgX as well as WT MotA. MotA A201, the target of the other MotA suppressor mutation, is predicted to occur at a transmembrane-cytoplasm interface (see Fig. S4A in the supplemental material). The MotA_A201V_ suppressor mutant restored motility to the Δ*flgX* mutant but at a reduced level compared to MotA_H138Y_. FtsH usually recognizes target proteins for degradation via nonpolar regions of approximately 20 amino acids in length at the N or C termini of proteins without a strictly defined sequence (53–56). H138 and A201 of C. jejuni MotA are positioned at more central positions of the protein. Thus, we do not anticipate that either H138Y or A201V would have disrupted a proteolytic site for FtsH. Instead, these point mutations may have altered the structure of MotA or the MotA_4_MotB_2_ heterohexameric complex such that MotA is more resistant to unfolding and recognition by FtsH. H138 is not conserved in *Salmonella* MotA, but this residue is naturally a tyrosine in H. pylori MotA (Fig. S4B). This natural alteration of MotA in H. pylori may negate the need of this epsilonproteobacterium to possess a FlgX ortholog to preserve stator integrity.

While C. jejuni Δ*flgX* displayed a 10,000-fold defect in commensal colonization of the chick ceca, the simple alteration of H138 of MotA to a tyrosine that occurred in one of the suppressor mutants restored colonization levels 1,000-fold for the Δ*flgX* mutant. Furthermore, the colonization capacity of C. jejuni Δ*flgX motA*_H138Y_ was only 10-fold lower than that WT C. jejuni. Due to this nearly complete restoration of stator protein levels, motility, and colonization, we think FlgX likely does not have other significant *in vivo* roles for C. jejuni outside maintaining stability of stator complexes. The modest reduction in *in vitro* motility conferred by MotA_H138Y_ compared to WT MotA may have contributed to the 10-fold colonization defect observed in the Δ*flgX mot*A_H138Y_ mutant relative to WT C. jejuni.

Our work has identified a unique role for FlgX in C. jejuni as a chaperone for the stator units of a bacterial flagellum. The employment of FlgX to maintain a sufficient level of stator units to power flagellar rotation represents another evolutionary adaptation of the C. jejuni flagellar motor and of bacterial flagellar motors in general. The maintenance of the integrity of stator units via FlgX, along with the evolutionary adaptations in the flagellar motor structure due to the incorporation of disk scaffolds that integrate more stator units and position them at a greater radial distance to increase torque, demonstrates the exquisite ability of C. jejuni to adapt and construct a high-torque motor to facilitate flagellar motility.

## MATERIALS AND METHODS

### Bacterial strains and plasmids.

The C. jejuni 81-176 strains used in this study are described in Table 2. All plasmids used or constructed for studies in this work are described in Table 3. Unless otherwise indicated, C. jejuni was grown from freezer stocks on Mueller-Hinton (MH) agar under microaerobic conditions (85% N_2_, 10% CO_2_, 5% O_2_) at 37°C for 48 h and then restreaked on MH agar and grown for 16 h under identical conditions prior to each experiment. Antibiotics were added to MH media as required at the following concentrations: 10 μg/ml trimethoprim; 15 μg/ml chloramphenicol; 30 μg/ml cefoperazone; or 0.5, 1, 2, or 5 mg/ml streptomycin. All C. jejuni strains were stored at −80°C in a 85% MH broth–15% glycerol solution. Escherichia coli DH5α and DH5/pRK212.1 were grown on Luria-Bertani (LB) agar or in LB broth containing 100 μg/ml ampicillin, 15 μg/ml chloramphenicol, or 12.5 μg/ml tetracycline as appropriate. All E. coli strains were stored at −80°C in a 80% LB broth–20% glycerol solution.

**TABLE 2:** Bacterial strains used in this study

Strain	Genotype	Source or reference
Escherichia coli strains		
DH5α	*supE44* Δ*lacU169* (φ80*lacZ*DM15) *hsdR17 recA1 endA1 gyrA96 thi-1 relA1*	Invitrogen
DH5α/pRΚ212.1	DH5α with conjugation transfer element	66
DAR5220	DH5α/pRΚ212.1 and pDAR5220	This study
DAR5266	DH5α/pRΚ212.1 and pDAR5226	This study

*Salmonella* strain		
IR715	Nalidixic acid-resistant spontaneous mutant of Salmonella enterica serovar Typhimurium strain 14028S	67

Campylobacter jejuni strains		
DRH212	81-176 *rpsL*^Sm^	58
DRH2469	81-176 *rpsL*^Sm^ *fliG*::*cat-rpsL*	68
DRH2508	81-176 *rpsL*^Sm^ Δ*fliG*	This study
DAR981	81-176 *rpsL*^Sm^ Δ*pflB*	3
DAR1066	81-176 *rpsL*^Sm^ Δ*motB*	3
DAR1131	81-176 *rpsL*^Sm^ Δ*motA*	3
DAR2060	81-176 *rpsL*^Sm^ *flgX*::*cat-rpsL*	This study
DAR2115	81-176 *rpsL*^Sm^ Δ*motB*/pDAR2053	This study
DAR2340	81-176 *rpsL*^Sm^ Δ*flgX*; in-frame deletion of codons 6-153	This study
DAR4064	81-176 *rpsL*^Sm^ Δ*motA*/pECO102	This study
DAR4329	81-176 *rpsL*^Sm^ Δ*motA*/pDAR4319	This study
DAR4781	81-176 *rpsL*^Sm^ Δ*motB*/pECO102	This study
DAR4803	81-176 *rpsL*^Sm^ Δ*flgX*; in-frame deletion of codons 101 to 153	This study
DAR4926	81-176 *rpsL*^Sm^ Δ*motAB*	This study
DAR4977	81-176 *rpsL*^Sm^ Δ*motAB*/pECO102	This study
DAR5007	81-176 *rpsL*^Sm^ Δ*flgX motA*::*cat-rpsL*	This study
DAR5020	81-176 *rpsL*^Sm^ Δ*flgX*/pDAR5009	This study
DAR5024	81-176 *rpsL*^Sm^ Δ*flgX*/pDAR5010	This study
DAR5026	81-176 *rpsL*^Sm^ Δ*flgX*/pDAR5011	This study
DAR5031	81-176 *rpsL*^Sm^ Δ*flgX*/pECO102	This study
DAR5040	81-176 *rpsL*^Sm^ Δ*motA flgX*::*cat-rpsL*	This study
DAR5110	81-176 *rpsL*^Sm^ Δ*flgX* Δ*motA*	This study
DAR5112	81-176 *rpsL*^Sm^ Δ*flgX motA*_H138Y_	This study
DAR5126	81-176 *rpsL*^Sm^ Δ*flgX motA*_H138Y_/pDAR5010	This study
DAR5133	81-176 *rpsL*^Sm^ Δ*flgX* Δ*motA*/pDAR5010	This study
DAR5148	81-176 *rpsL*^Sm^ *motA*_H138Y_	This study
DAR5158	81-176 *rpsL*^Sm^ Δ*motAB flgX*::*cat-rpsL*	This study
DAR5161	81-176 *rpsL*^Sm^ Δ*motB flgX*::*cat-rpsL*	This study
DAR5201	81-176 *rpsL*^Sm^ Δ*fliG flgX*::*cat-rpsL*	This study
DAR5203	81-176 *rpsL*^Sm^ Δ*pflB flgX*::*cat-rpsL*	This study
DAR5223	81-176 *rpsL*^Sm^ Δ*flgX* Δ*motB*	This study
DAR5236	81-176 *rpsL*^Sm^ Δ*motAB*/pDAR5220	This study
DAR5240	81-176 *rpsL*^Sm^ Δ*flgX* Δ*motAB*	This study
DAR5242	81-176 *rpsL*^Sm^ Δ*flgX* Δ*motB*/pDAR5010	This study
DAR5250	81-176 *rpsL*^Sm^ Δ*flgX* Δ*motAB*	This study
DAR5254	81-176 *rpsL*^Sm^ Δ*flgX* Δ*motAB*/pDAR5010	This study
DAR5260	81-176 *rpsL*^Sm^ Δ*flgX* Δ*motAB* /pDAR5220	This study
DAR5280	81-176 *rpsL*^Sm^ Δ*motAB*/pDAR5266	This study
DAR5301	81-176 *rpsL*^Sm^ Δ*flgX* Δ*motAB*/pDAR5266	This study
CRG479	81-176 *rpsL*^Sm^/pDAR964	69
MB1225	81-176 *rpsL*^Sm^ *motA*::*cat-rpsL*	3

**TABLE 3:** Plasmids used in this study

Plasmid	Genotype	Source or reference
pUC19	Amp^r^; general cloning vector^*a*^	New England Biolabs
pECO102	Cat^r^; Escherichia coli*-*Campylobacter jejuni shuttle vector containing *cat* promoter for expression of genes for complementation	70
pDRH265	Source of *cat-rpsL* cassette	58
pDRH3330	pUC19 containing *motA*::*cat-rpsL*	3
pDAR964	Cat^r^; Escherichia coli*-*Campylobacter jejuni shuttle vector containing *cat* promoter with an in-frame N-terminal FLAG sequence for expression of genes for complementation	69
pDAR2033	pUC19 with DNA fragment to create in-frame deletion of codons 6 to 153 of *flgX* with 0.5 kb of upstream and downstream sequence cloned into the BamHI site	This study
pDAR2041	pUC19 with DNA fragment harboring the *flgWX* locus cloned into the BamHI site	This study
pDAR2047	SmaI *cat-rpsL* cassette cloned into the SwaI site of *flgX* in pDAR2041	This study
pDAR2048	Same as pDAR2047	This study
pDAR2053	pDRH719 with *motB* from codon 2 to the penultimate codon fused at the 3′ end to a FLAG tag with a stop codon cloned into the BamHI and PstI site of pDRH719	This study
pDRH2439	pUC19 with DNA fragment to create in-frame deletion of codons 3–314 of *fliG* with 0.75 kb of upstream and downstream sequence cloned into the BamHI site	This study
pDAR3369	pUC19 with DNA fragment to create in-frame deletion of codons 101 to 153 of *flgX* with 0.5 kb of upstream and downstream sequence cloned into the BamHI site	This study
pDAR4319	pDAR964 with *motA* from codon 2 to the stop codon cloned into the BamHI site of pDAR964	This study
pDAR4918	pUC19 with in-frame fusion of *motA* codons 1–11 to the last 6 codons of *motB* to delete most of the coding regions of *motA* and *motB*	This study
pDAR5002	pUC19 with the *motAB* locus containing *motA*_H138Y_ cloned into the EcoRI site	This study
pDAR5009	pECO102 with *flgX* coding sequence from codon 2 to the stop codon cloned into the BamHI site	This study
pDRH5010	pDAR964 with *flgX* coding sequence from codon 2 to the stop codon cloned into the BamHI site	This study
pDRH5011	pECO102 with *flgX* coding sequence from codon 2 to the penultimate codon fused at the 3′ end to a FLAG tag with a stop codon cloned into the BamHI site of pDRH719	This study
pDAR5266	pECO102 with Campylobacter jejuni *motAB* coding sequence from codon 2 of *motA* through the penultimate codon of *motB* fused at the 3′ end of *motB* to a FLAG tag with a stop codon cloned into the BamHI site	This study
pDAR5220	pECO102 with Salmonella enterica *motAB* coding sequence from codon 2 of *motA* through the penultimate codon of *motB* fused at the 3′ end of *motB* to a FLAG tag with a stop codon cloned into the BamHI site	This study
pSMS511	pQE30::*flgP*	60

a Amp, ampicillin.

### Construction of C. jejuni mutants.

C. jejuni mutants were constructed by electroporation of plasmid DNA or natural transformation of *in vitro*-methylated plasmid DNA following previously described methods (57, 58). All plasmids were constructed by ligation of DNA fragments into plasmids by the use of T4 DNA ligase or Gibson assembly mastermix (New England Biolabs).

To delete *flgX* (*Cjj81176_0199*) from the C. jejuni 81-176 Sm^r^ chromosome, a fragment containing the *flgWX* locus (*Cjj81176_0198* and *Cjj81176_0199*) with ∼500 bases of flanking sequence was amplified and cloned into the BamHI site of pUC19 to create pDAR2041. A SmaI-digested *cat-rpsL* cassette from pDRH265 was ligated into a SwaI site of *flgX*, creating two identical plasmids, pDAR2047 and pDAR2048. pDAR2047 was then electroporated into C. jejuni 81-176 *rpsL*^Sm^ (DRH212) to interrupt *flgX* on the chromosome with the *cat-rpsL* cassette. Transformants were recovered on MH agar containing chloramphenicol. Mutations were verified by colony PCR to obtain DAR2060 (strain 81-176 *rpsL*^Sm^ *flgX*::*cat-rpsL*). Due to ambiguity regarding the start site for *flgX*, two Δ*flgX* strains were constructed in which in-frame deletions of portions of *flgX* were created on the chromosome. Plasmids containing deletion constructs were created by designing primers with 5′ BamHI sites to amplify from the 81-176 genome two DNA fragments with ∼500 nucleotides upstream and downstream of the portion of *flgX* to be deleted. The DNA fragments were then fused together by PCR, followed by cloning into BamHI-digested pUC19. pDAR2033 contains a deletion of codons 6 to 153 of *flgX*, and pDAR3369 contains a deletion of codons 101 to 153. These plasmids were then introduced into DAR2060, and transformants were recovered on MH agar with streptomycin and then screened for chloramphenicol sensitivity. Colony PCR verified the correct deletion mutants to result in DAR2340 (strain 81-176 *rpsL*^Sm^ Δ*flgX* containing a deletion of codons 6 to 153) and DAR4803 (strain 81-176 *rpsL*^Sm^ Δ*flgX* containing a deletion of codons 101 to 153).

DNA fragments with ∼750 nucleotides upstream and downstream of the portion of *fliG* to be deleted were amplified from the 81-176 genome and then fused together by PCR. The fragment was then cloned into the BamHI site of pUC19 to create pDRH2439. This plasmid was electroporated into DRH2469 (strain 81-176 *rpsL*^Sm^ *fliG*::*cat-rpsL*). Transformants were selected on MH agar containing streptomycin and screened for chloramphenicol sensitivity. Transformants with the correct deletion were verified by colony PCR to obtain DRH2508 (strain 81-176 *rpsL*^Sm^ Δ*fliG*).

For construction of a strain 81-176 *rpsL*^Sm^ Δ*motAB* mutant, DNA fragments of the *motAB* region were amplified and then assembled into EcoRI-digested pUC19 to result in pDAR4918, which contained a fusion of the first 11 codons of *motA* to the last 6 codons of *motB*. This plasmid was transformed into MB1225 (strain 81-176 *rpsL*^Sm^ *motA*::*cat-rpsL*), and transformants were recovered on MH agar with streptomycin. Creation of DAR4926 (strain 81-176 *rpsL*^Sm^ Δ*motAB*) was confirmed by colony PCR. To delete *flgX* from C. jejuni mutant strains, pDAR2047 or pDAR2048 was introduced into DAR981, DAR1131, DAR1066, DAR4926, and DRH2508. Transformants were recovered on MH agar containing chloramphenicol and verified by colony PCR, yielding DAR5040 (strain 81-176 *rpsL*^Sm^ Δ*motA flgX*::*cat-rpsL*), DAR5161 (strain 81-176 *rpsL*^Sm^ Δ*motB flgX*::*cat-rpsL*), DAR5158 (strain 81-176 *rpsL*^Sm^ Δ*motAB flgX*::*cat-rpsL*), DAR5201 (strain 81-176 *rpsL*^Sm^ Δ*fliG flgX*::*cat-rpsL*), and DAR5203 (Δ*pflB flgX*::*cat-rpsL* mutant). Deletion of *flgX* was then accomplished as previously described by introducing pDAR2033 into DAR5040, DAR5161, and DAR5158 to yield DAR5110 (strain 81-176 *rpsL*^Sm^ Δ*motA* Δ*flgX*), DAR5223 (strain 81-176 *rpsL*^Sm^ Δ*motB* Δ*flgX*), and DAR5240 and DAR5250 (both representing strain 81-176 *rpsL*^Sm^ Δ*motAB* Δ*flgX*), respectively.

### Construction of plasmids for complementation.

Plasmids to complement Δ*flgX* strains were constructed by amplifying *flgX* from codon 2 to the stop codon (with and without a FLAG tag attached to the 3′ end prior to the stop codon) from the 81-176 genome, followed by assembly into BamHI-digested pECO102 or pDAR964 to yield pDAR5009, pDAR5011, and pDAR5010. Plasmids to complement Δ*motA* and Δ*motB* mutants were created by amplifying the coding sequence from codon 2 to the stop codon of *motA* or codon 2 to the penultimate codon of *motB* with DNA for a FLAG tag, and a stop codon was added to the 3′ end. These fragments were then cloned into the BamHI site of pDAR964 and the BamHI and PstI sites of pECO102, resulting in plasmids pDAR4319 and pDAR2053, respectively. Plasmids to express the *motAB* loci from C. jejuni 81-176 or S. enterica serovar Typhimurium IR715 were created by amplifying the coding region of *motA* and *motB*, which are adjacent to each other on the respective chromosomes. The *motAB* region from codon 2 of *motA* through the penultimate codon of *motB* was amplified with primers that added DNA for a FLAG tag to the 3′ end of *motB* followed by a stop codon. These primers also contained 5′ BamHI sites for introduction into BamHI-digested pECO102, resulting in plasmids pDAR5266 and pDAR5220. All plasmids were transformed into E. coli DH5α and pRK212.1, which served as donor strains for conjugation into C. jejuni. Plasmids were then conjugated into C. jejuni strains as previously described (59).

### Motility assays.

After standard growth for 16 h on MH agar, C. jejuni strains were resuspended from plates in MH broth and diluted to an optical density at 600 nm (OD_600_) of 0.8. Each bacterial strain was stabbed into semisolid MH motility media containing 0.4% agar using an inoculation needle and then incubated for 24 to 48 h at 37°C under microaerobic conditions. When appropriate, motility agar that contained chloramphenicol was used to maintain plasmids for in *trans* complementation of mutants.

### Generation of antisera.

For generation of anti-FlgP rabbit antiserum, recombinant 6×His-tagged FlgP was purified as previously described (60). Purified recombinant protein was used to immunize rabbits for generation of antiserum by a commercial vendor (Cocalico Biologicals).

### Protein preparation and immunoblotting analyses.

Whole-cell lysates (WCL) were prepared as previously described (61). Fractionation of C. jejuni into subcellular compartments for analysis of protein localization was performed as previously described (62), with slight modifications. Briefly, after growth, C. jejuni strains were resuspended in 1× phosphate-buffered saline (PBS) and diluted to an OD_600_ of 0.8 to obtain equal densities of bacteria for all cultures before any fractionation procedures were performed. For WCL, 1 ml of bacterial culture was pelleted, washed once in PBS, and resuspended in 50 μl 1× SDS-PAGE loading buffer. To generate periplasmic and cytoplasmic fractions, 20 ml of bacterial culture was washed twice with 2 ml of PBS containing 0.1% gelatin (PBSG) and then resuspended in 2 ml of PBSG containing 20 mg/ml polymyxin B sulfate (Sigma) to compromise the outer membrane and release the periplasmic contents. After centrifugation, the supernatant was saved as the periplasmic fraction, and the recovered pellets consisted of whole spheroplasts. The spheroplast preparation was resuspended in 1 ml 10 mM HEPES and sonicated. Following centrifugation, the supernatants representing soluble cytoplasmic proteins were recovered. For the inner and outer membrane protein fractions, 5-ml aliquots of bacterial culture were pelleted and washed once with 1 ml of 10 mM HEPES. Bacteria were then resuspended in 1 ml of 10 mM HEPES and sonicated. Insoluble material representing total membrane proteins were recovered after centrifugation for 30 min at 16,000 × *g*. Membranes were resuspended in 10 mM HEPES containing 1% *N*-lauroylsarcosine sodium salt to solubilize inner membrane proteins. The soluble inner membrane proteins were separated from the insoluble outer membrane proteins by centrifugation for 30 min at 16,000 × *g*.

For analysis of proteins from E. coli whole-cell lysates, total membrane, and soluble fractions, overnight cultures of E. coli were inoculated at a 1:20 dilution and grown at 37°C with shaking to an OD_600_ of 0.8. For whole-cell lysates, 1 ml of culture was collected by centrifugation and resuspended in 50 ml of 1 × SDS-PAGE loading buffer. For separation of soluble and total membrane fractions, 5 ml of culture was collected by centrifugation, washed once in 1 ml of 10 mM HEPES, and then sonicated. After sonication, samples were centrifuged for 30 min at full speed to pellet total membrane fractions, with the supernatant representing the soluble proteins from the cytoplasm and periplasm.

SDS-PAGE for separation of proteins and for transfer to membranes for immunoblotting analysis was performed by standard procedures. Protein samples of C. jejuni or E. coli whole-cell lysates or cellular fractions of C. jejuni were loaded from standardized culture densities at equal levels. Primary antisera were used at the following dilutions: FliF M204, 1:5,000 (63); MotA GP155, 1:5,000 (3); MotB GP140, 1:5,000 (3); RpoA M251, 1:2,000 (61); Cjj0382 M17, 1:2,000 (62); FlgP R702, 1:20,000; PflB GP143, 1:2,000 (3); anti-FLAG M2, 1:2,000 (Sigma); RpoD, 1:10,000 (Thermo Fisher). Blots were incubated with primary sera for 1 to 2 h. Secondary horseradish peroxidase (HRP)-conjugated goat antibody to detect each primary antibody was diluted 1:10,000 and incubated for 1 h. Immunoblots were developed by using a Western Lightning Plus ECL kit (Perkin-Elmer).

### Semiquantitative real-time PCR analysis.

WT C. jejuni and isogenic mutant strains were suspended from MH agar plates after growth, total RNA was extracted with RiboZol (Amresco), and RNA was treated with DNase I (Invitrogen). RNA was diluted to a concentration of 5 ng/μl before analysis. Semiquantitative real-time PCR (qRT-PCR) was performed using a 7500 real-time PCR system (Applied Biosystems) with *gyrA* mRNA detection as an endogenous control. For measurements of *motA* and *motB* mRNA transcript levels, strain CRG479 (strain 81-176 *rpsL*^Sm^/pDAR964) served as the control to determine relative gene expression levels in isogenic mutants. For measurements of *ftsH* mRNA transcript levels, DAR4803 (strain 81-176 *rpsL*^Sm^ Δ*flgX* with deletion of codons 101 to 153) served as the control to determine relative gene expression levels in isogenic suppressor mutants.

### *In vivo* immunoprecipitation of C. jejuni proteins.

Coimmunoprecipitation of proteins from C. jejuni strains expressing FLAG-tagged FlgX proteins was performed as previously described (63), with a few modifications. Briefly, after growth on MH agar with appropriate antibiotics for 16 h at 37°C under microaerobic conditions, bacteria from two plates of growth were suspended in PBS and collected by centrifugation. Bacteria were resuspended in 2 ml PBS and then cross-linked by the addition of formaldehyde (0.1% final concentration) for 30 min at room temperature with shaking, followed by quenching with 0.4 ml of 1 M glycine for 10 min. Bacteria were then collected by centrifugation and then disrupted by osmotic lysis with sequential addition of 0.5 ml 200 mM Tris (pH 8), 1 ml 200 mM Tris (pH 8), 1 M sucrose, 0.1 ml 10 mM EDTA, 0.1 ml 10 mg/ml lysozyme, 3 ml double-distilled water (dH_2_O), and 0.3 ml 100 mM phenylmethylsulfonyl fluoride (PMSF) (64). After incubation on ice for 15 min, 5 ml of lysis solution (50 mM Tris [pH 8.0], 10 mM MgCl_2_, 2% Triton X-100) was added. Samples were incubated on ice for 45 min and then centrifuged at 16,000 × *g* for 20 min. A 30-μl volume of anti-FLAG M2 affinity gel resin was added to the supernatant, and the reaction mixture was then incubated at 4°C overnight with agitation. The resin was pelleted by centrifugation at 4°C for 10 min at 6,000 × *g* followed by 3 washes with radioimmunoprecipitation assay (RIPA) buffer (50 mM Tris [pH 8.0], 150 mM NaCl, 0.1% SDS, 0.5% sodium deoxycholate, 1% Triton X-100). For immunoblotting, the resin was resuspended in 70 μl SDS-PAGE loading buffer, boiled for 5 min, and analyzed by 10% SDS-PAGE and immunoblotting with specific antisera.

### C. jejuni Δ*flgX* suppressor analysis.

To isolate and identify suppressor mutants that partially restored the motility phenotype of C. jejuni Δ*flgX*, DAR2340 (strain 81-176 *rpsL*^Sm^ Δ*flgX*, which contains a large deletion of *flgX* from codons 6 to 153) and DARH4803 (strain 81-176 *rpsL*^Sm^ Δ*flgX*, which contains a smaller deletion of *flgX* from codons 101 to 153) were resuspended in MH broth from plates after growth to an OD_600_ of 0.8 and stabbed into MH semisolid motility agar. Bacteria were then incubated for up to 10 days at 37°C under microaerobic conditions. Potential suppressor mutants were identified as motile flares that emanated from the point of inoculation of different motility stabs. A small agar plug from the leading edge of motile flares was recovered and subjected to vortex mixing in MH broth. Suppressor mutants from each plug were isolated by plating serial dilutions on MH agar. Three isolates from each agar plug were saved.

Genomic DNAs from a parental C. jejuni Δ*flgX* strain (DRH4803) and from corresponding suppressor mutant strains were prepared as previously described (65). Briefly, isolates were grown on Mueller-Hinton agar plates supplemented with 10 μg/ml trimethoprim at 37°C under microaerobic conditions. Bacteria were harvested, and genomic DNA was isolated using a Qiagen DNeasy blood and tissue kit. The resulting DNA was treated with RNase If (New England BioLabs) and cleaned using a Zymo genomic DNA clean and concentrator kit. Prior to submission for sequencing, the DNA samples were run on a 1.0% agarose gel to check DNA integrity. Genomic DNA from the experiments described above was used to generate bar-coded Bioo NEXTflex DNA libraries at the Indiana University Center for Genomics and Bioinformatics. These libraries were cleaned and verified using an Agilent 2200 TapeStation before pooling and sequencing on the Illumina NextSeq platform were performed. Paired-end reads were demultiplexed before the analysis described below was performed.

Reads obtained from the parent (DAR4803) and suppressor mutant genomes were mapped to the C. jejuni 81-176 reference genome (GCA_000015525.1) using Geneious R10.2.6.

Single nucleotide polymorphisms (SNPs) with a minimum variant frequency of 20% were identified in regions of the parent and suppressor mutant genomes with at least 5× coverage. Individual SNPs identified in suppressor mutant genomes were compared to those identified within the parent genome. SNPs that were unique to the suppressor mutant genomes were presumed to be associated with the suppressor mutant phenotypes and investigated further. Identification of other suppressor mutants for which genomic sequencing was not applied involved PCR amplification of one or more suspected genes and then sequencing of PCR products.

To reconstitute the *motA*_H138Y_ suppressor allele identified in DAR4803 S10 on the chromosome of WT C. jejuni, DNA fragments containing the *motAB* locus that encode the H138Y mutation with approximately 0.5 kb of upstream or downstream sequence were amplified. The fragments were then assembled into EcoRI-digested pUC19 to yield pDAR5002. pDRH3330 was introduced into DAR2340 (strain 81-176 *rpsL*^Sm^ Δ*flgX*), and transformants were obtained on MH agar containing chloramphenicol. Colony PCR verified creation of DAR5007 (strain 81-176 *rpsL*^Sm^ Δ*flgX motA*::*cat-rpsL*). *motA*::*cat-rpsL* was replaced with *motA*_H138Y_ in MB1225 (strain 81-176 *rpsL*^Sm^ *motA*::*cat-rpsL*) and DAR5007 by introduction of pDAR5002 and selection of transformants on MH agar with streptomycin. Potential transformants were screened for chloramphenicol sensitivity and were then verified by colony PCR and sequencing to obtain DAR5148 (strain 81-176 *rpsL*^Sm^ *motA*_H138Y_) and DAR5112 (strain 81-176 *rpsL*^Sm^ Δ*flgX motA*_H138Y_).

### Chick colonization assays.

All uses of animals in this work were approved by the IACUC at the University of Texas Southwestern Medical Center. The ability of wild-type C. jejuni or mutant strains to colonize the ceca of chicks after oral inoculation was determined as previously described (49). Briefly, fertilized chicken eggs (SPAFAS) were incubated for 21 days at 37.8°C with appropriate humidity and rotation in a Sportsman II model 1502 incubator (Georgia Quail Farms Manufacturing Company). Approximately 12 to 36 h after hatching, chicks were orally infected with 100 μl of PBS containing approximately 10^4^ CFU of WT C. jejuni or an isogenic mutant strain. Strains were prepared for oral gavage by resuspension from MH agar after growth and dilution in PBS to an OD_600_ of 0.4 followed by serial dilution to obtain the appropriate inoculum for oral gavage of chicks. The CFU count of the inoculum was determined by serial dilution on MH agar. At day 14 postinfection, chicks were sacrificed and the cecal contents were recovered, weighed, and suspended in PBS to 0.1 g cecal content/ml PBS. Serial dilutions were spread on MH agar containing TMP and cefoperazone. Bacteria were grown for 72 h at 37°C under microaerobic conditions and then counted to determine the CFU per gram of cecal content for each chick. Approximately 50 recovered colonies per chick were stabbed into MH motility agar to determine whether suppressors had developed during *in vivo* growth. Motility was assessed after 24 to 30 h of incubation at 37°C under microaerobic conditions. Statistical analyses were performed by the use of the Mann-Whitney U test, with statistically significant differences between wild-type and mutant strains indicated with *P* values of <0.05.

### Data availability.

All data and descriptions of the methodologies employed are available upon request. Genomic sequences are available at NCBI as part of Bioproject PRJNA543870, with individual genomic sequences assigned the following accession numbers: DAR4803 (SRR9141309); DAR4803 S1 (SRR9141310); DAR4803 S4 (SRR9141311); DAR4803 S6 (SRR9141312); DAR4803 S7 (SRR9141313); DAR4803 S8 (SRR9141314); DAR4803 S9 (SRR9141315); and DAR4803 S10 (SRR9141316).
