# Supplementary figures and images for: A Chaperone for the Stator Units of a Bacterial Flagellum
Source: mBio. 2019 Aug 6;10(4):e01732-19. doi: 10.1128/mBio.01732-19 (PMC6686046; doi:10.1128/mBio.01732-19)

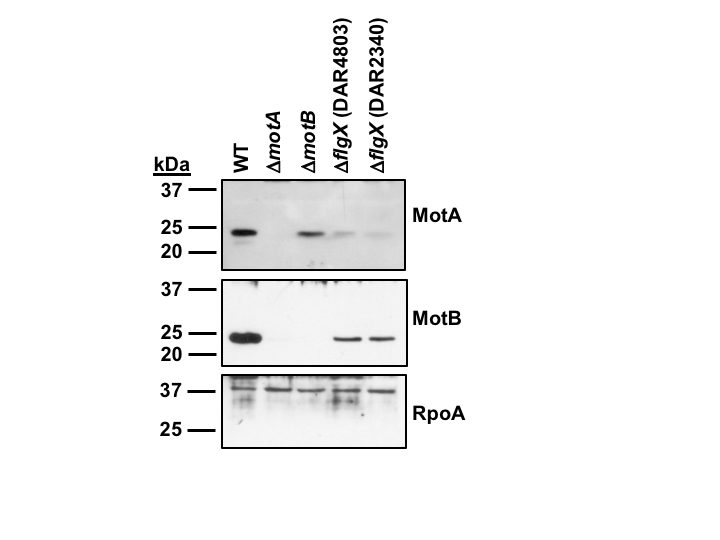

Supplement: FIG S1 [file mBio.01732-19-sf001.tif]

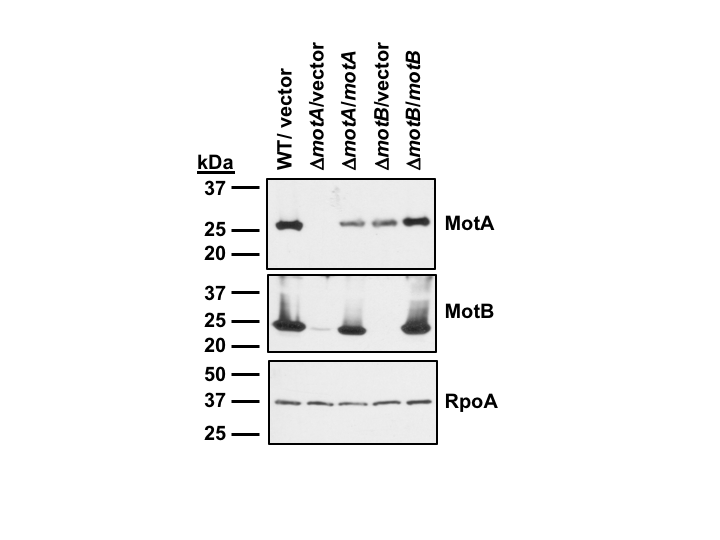

Supplement: FIG S2 [file mBio.01732-19-sf002.tif]

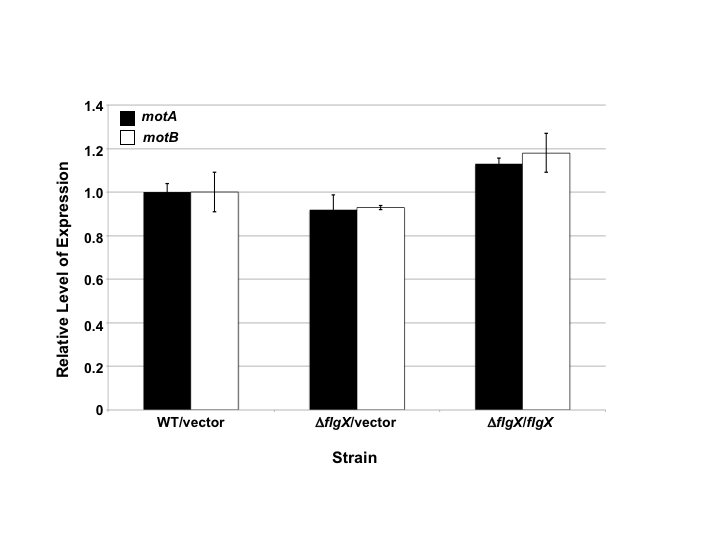

Supplement: FIG S3 [file mBio.01732-19-sf003.tif]

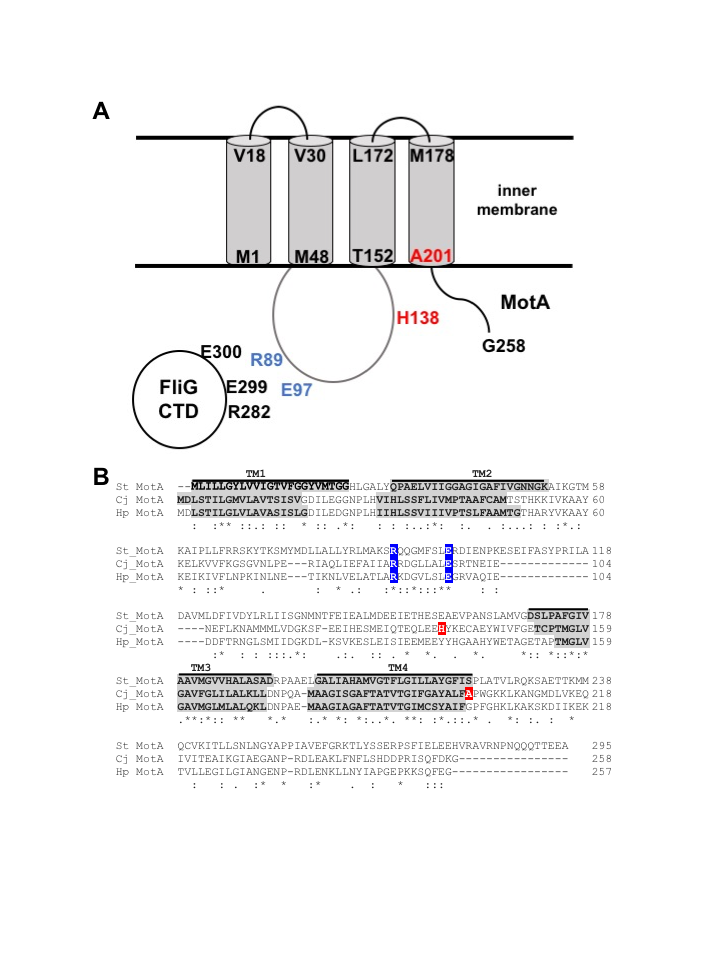

Supplement: FIG S4 [file mBio.01732-19-sf004.tif]
